# Metastatic colorectal cancer and severe hypocalcemia following irinotecan administration in a patient with X-linked agammaglobulinemia: a case report

**DOI:** 10.1186/s12881-019-0880-1

**Published:** 2019-09-12

**Authors:** Mingming Li, Wei Chen, Xiaomeng Sun, Zhipeng Wang, Xun Zou, Hua Wei, Zhan Wang, Wansheng Chen

**Affiliations:** 1 Department of Pharmacy, Changzheng Hospital, Secondary Military Medical University, Shanghai, China; 2 Institutes of Biomedical Sciences, Fudan University, Shanghai, China; 3 Department of Oncology, Changzheng Hospital, Secondary Military Medical University, Shanghai, China

**Keywords:** X-linked agammaglobulinemia, Hypocalcemia, Whole exome sequencing, Therapeutic drug monitoring, And irinotecan

## Abstract

**Background:**

X-linked agammaglobulinemia (XLA) is a primary immunodeficiency disorder caused by germline mutations in the Bruton tyrosine kinase (*BTK*) gene on X chromosome. These mutations disturb B-cell development, decrease immunoglobulin levels, increase susceptibility to infection or neoplasms, and increase the risk of developing colorectal cancer (CRC). For occasional cases of CRC have been reported in XLA patients, low levels of B lymphocytes and immunoglobulins induced by congenital immune disorder make them more susceptible to drug-related toxicities (DRT). Therefore, gene sequencing, therapeutic drug monitoring and any possible measurement to predict DRT should be considered before determining the course of chemotherapy for XLA patients with CRC.

**Case presentation:**

In this study, we reported a 21-year-old male who developed metastatic CRC in the context of XLA. Since the whole exome sequencing and therapeutic drug monitoring did not reveal any predictive markers of DRT, we applied standard first-line chemotherapy to the patient. However, progressive disease occurred after the fifth treatment cycle. Therefore, the administration of oxaliplatin was changed to irinotecan as second-line therapy. After that, the patient firstly suffered from severe hypocalcemia and eventually died due to metastatic CRC after the eighth treatment cycle. The overall survival time was 7.5 months.

**Conclusions:**

This study reported the first written record of a Chinese XLA patient with metastatic CRC and severe hypocalcemia. Whole exome sequencing and bioinformatic analysis indicated the somatic mutations in *ABCA6*, *C6* and *PAX3* genes might contribute to the early-onset and metastasis CRC. Besides, a number of germline mutations in genes related to calcium metabolism (*CACNA2D4, CD36,* etc.) and the administration of irinotecan were speculated to be the causes of severe hypocalcemia. We therefore suggested that in order to avoid severe DRT, clinicians should take genetic background and therapeutic drug monitoring into consideration while planning chemotherapy treatment for XLA patients with CRC.

**Electronic supplementary material:**

The online version of this article (10.1186/s12881-019-0880-1) contains supplementary material, which is available to authorized users.

## Background

X-linked agammaglobulinemia (XLA) is an X-linked inherited disease caused by genetic mutations in the Bruton tyrosine kinase (*BTK*) gene [1, 2], which suppress the development of mature B lymphocytes. The human *BTK* gene encompasses 37.5 kb containing 19 exons. BTKbase is an up-to-date database compiling 1796 entities showing 917 unique *BTK* mutations from 1749 individuals, two thirds of which are from unrelated families, while one third are believed to be sporadic cases [3]. These mutations are found throughout the entire *BTK* gene sequence. The incidence of XLA varies between 1/200,000 and 1/20,000,000 in Western countries, whereas it has not been calculated in China yet [4]. Based on estimation, there should be more than 1000 cumulative XLA cases below 14 years of age [5].

XLA patients are characterized by insufficient number of normal circulating B lymphocytes and immunoglobulins [6]. The concurrent hallmark symptoms and complications of XLA include lower respiratory tract infection (bronchitis/pneumonia), otitis media, persistent diarrhea and skin infections [7, 8]. XLA patients are also susceptible to certain types of cancer including colorectal cancer (CRC) [9–15]. According to the National Comprehensive Cancer Network (NCCN) guidelines for colon cancer and rectal cancer, 5-fluorouracil (5-FU)-based drugs are recommended and commonly used for first-line chemotherapy [16]. But patients receiving 5-FU-based chemotherapy, alone or in a combination regimen, may experience drug-related toxicities (DRT) involving hand-foot syndrome, leukopenia, neutropenia, thrombocytopenia, diarrhea, nausea and vomiting [17]. Severe DRT not only leads to an early termination of chemotherapy but also causes safety issues. With previous evidence of lymphopenia being an independent factor associated with first-line chemotherapy induced hematologic toxicities in CRC patients [18, 19], we assume that XLA patients, who are characterized by low levels of B lymphocytes and immunoglobulins, are more likely to develop DRTs when they are diagnosed with CRC and receive chemotherapy [11]. Therefore, gene sequencing, therapeutic drug monitoring and any possible measurement to predict DRT should be considered before making chemotherapy regimens for XLA patients with CRC.

## Case presentation

### Presenting concerns

**Fig. 1:**
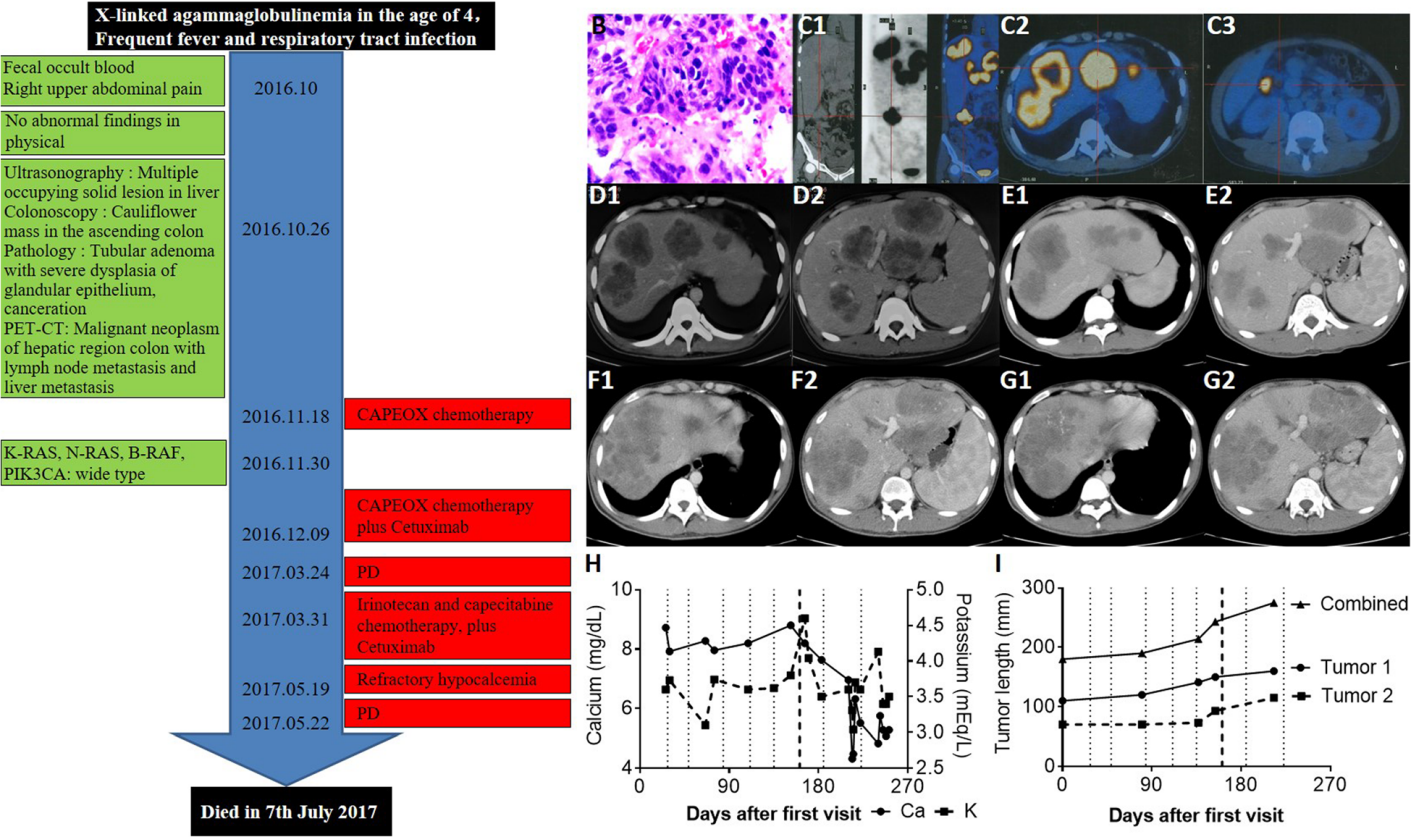
The timeline, diagnosis and progression focus. **a** The timeline of hospitalization: This patient was diagnosed with XLA at the age of 4. He suffered upper abdominal pain on Oct 2016, and was diagnosed with advanced colorectal cancer with liver metastasis by liver biopsy ((**b**), magnification, × 100) and PET-CT scan (**c**). Abdominal CT scans on Oct 21th 2016 (**d**) and Jan 9th 2017 (**e**) showed that the tumor was stabilized after the first two treatment cycles. However, abdominal CT scans on Mar 24th 2017 (**f**) and May 22th 2017 (**g**) showed metastatic tumor progressed. Blood calcium/potassium levels (**h**) and tumor load (**i**) were recorded throughout all treatment cycles. The dotted lines indicate the starting time of each chemotherapy treatment cycle. The dark dotted line indicates the sixth treatment cycle when oxaliplatin was replaced by irinotecan

A 21-year-old man with XLA was hospitalized for fecal occult blood, epigastric pain and bronchitis in 2016. The patient was not married. The timeline of hospitalization is shown in Fig. 1a.

### Clinical findings

This patient was diagnosed with XLA when he was 4 years old. He had no family history of XLA. Regular intravenous immunoglobulin (IVIG) replacement therapy was applied since the diagnosis. The admission physical examination found no positive symptoms of XLA.

### Diagnostic focus and assessment

Abdominal ultrasonography showed multiple hepatic parenchymal lesions, gallbladder stones, splenomegaly and a hypoechoic mass in the right lower abdomen. Colonoscopy showed a cauliflower-like mass in the ascending colon. Needle biopsy of focal liver lesions (Fig. 1b) and PET-CT (Fig. 1c) suggested metastatic adenocarcinoma. The Eastern Cooperative Oncology Group (ECOG) Performance Status was one [20].

Whole exome sequencing (WES) was conducted using blood and liver tumor tissue. It revealed 10 somatic (Table 1) and 200 germline SNVs, including one on the *BTK* gene (c.340_347del, p.F114delX115) (Additional file 2: Figure S1). Somatic mutation was absent in *KRAS, NRAS, BRAF, or PIK3CA*. There is also no mutation in genes related to efficacy or safety of 5-FU-based drugs (Additional file 3: Table S1).

**Table 1:** Somatic variants may initiate tumor development

SNP ID	Gene	Chrs	Gene Region	Function	Cancer-promoting gene	Diseases
rs188382333	ABCA6	17	exonic	nonsynonymous SNV	Y	Colorectal cancer, acute myeloid Leukemia
rs201064036	C6	5	exonic	nonsynonymous SNV	Y	C6 deficiency
rs79930314	CCDC144NL	17	exonic	Stop gain	N	Colorectal cancer; Renal cancer
rs41291550	CYP2C18	10	exonic	Stop gain	N	NA
rs1799931	NAT2	8	exonic	nonsynonymous SNV	N	Slow acetylation
rs192410865	NOX3	6	exonic	Stop gain	N	NA
NM_000438:exon2:c.232G>A:p.V78M	PAX3	2	exonic	nonsynonymous SNV	Y	Alveolar rhabdomyosarcomas, type1 and type3 Waardenburg syndrome
rs138133378	SCN7A	2	exonic	nonsynonymous SNV	N	NA
rs117153533	TC2N	14	exonic	nonsynonymous SNV	N	Colorectal cancer,
rs201277886	TOX	8	exonic	nonsynonymous SNV	N	Colorectal cancer, virus-associated hepatocellular cancer, gastric cancer, esophageal cancer

Each SNP was searched in the following databases to confirm its correlation with diseases and whether it belongs to a cancer-promoting gene: ClinVar, ICGC, COSMIC, HGMD and OMIM

### Therapeutic focus and assessment

**Fig. 2:**
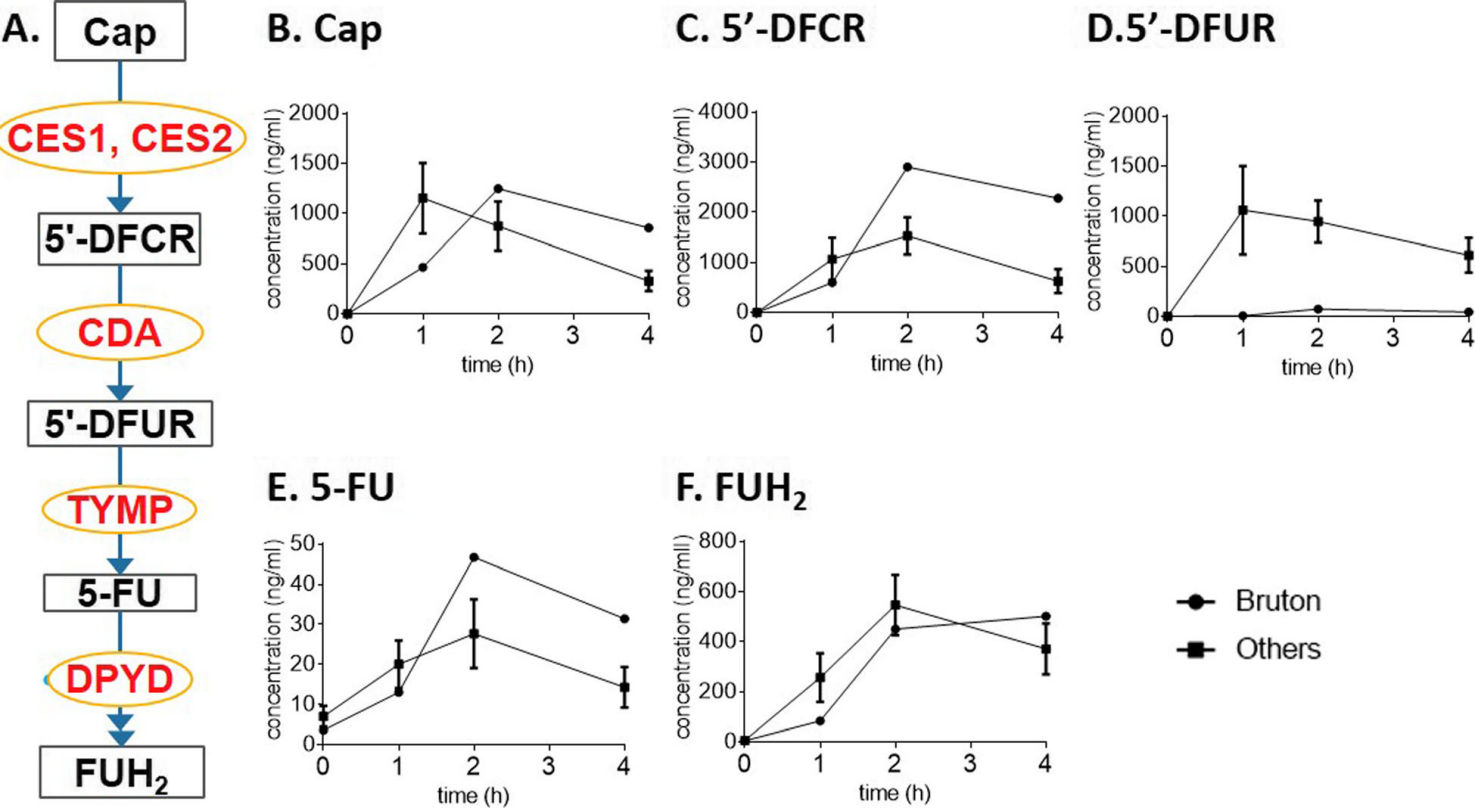
Therapeutic drug monitoring on Cap and its metabolites. **a** A simplified metabolic pathway of Cap. Metabolites and enzymes are indicated in black and red, respectively. Plasma concentrations of Cap (**b**) and its metabolites 5′-dFCR (**c**), 5′-dFUR (**d**), 5-FU (**e**), and FUH_2_ (**f**) were measured before and 1–4 h after the administration of Cap. The concentrations are presented as the mean ± SEM. Abbreviations: Cap, capecitabine; 5′-dFCR, 5′-deoxy-5-fluorocytidine; 5′-dFUR, doxifluridine; 5-FU, 5-fluorouracil; FUH_2_, 5-fluoro-5,6-dihydrouracil; CES, carboxylesterase; CDA, cytidine deaminase; TYMP, thymidine phosphorylase; and DPYD, dihydropyrimidine dehydrogenase

Standard first-line chemotherapy was started on Nov. 18th, 2016. The regimen is as follows: 130 mg/m^2^ oxaliplatin *iv* on day 1, 1000 mg/m^2^ capecitabine (Cap) twice *po* daily for 14 days, which was repeated every 3 weeks, and 500 mg/m^2^ cetuximab *iv* over 2 h on day 1 every 2 weeks. The tumor was stabilized after the first two treatment cycles (Fig. 1d-e), and the abdominal pain ceased, the abdominal lump was reduced, and CA-199 (from 1410 to 157 U/ml) and CEA (from 113.6 to 20.1 μg/L) levels decreased. During the second treatment cycle, TDM on Cap and its metabolites was conducted. Compared to 10 other random CRC patients without XLA, the XLA patient showed delayed Cmax of Cap, lower doxifluridine (5′-DFUR) level and higher 5-FU level (Fig. 2). These results suggested that the XLA patient had a slower ability to absorb Cap and increased thymidine phosphorylase (TYMP) activity. Since both low 5′-DFUR levels and induced TYMP activity are negatively associated with Cap-related toxicity [21, 22] and this patient did not show any sign of DRT, the original chemotherapy plan was not adjusted.

### Follow-up and outcomes

After the fifth treatment, the metastatic tumor progressed (PFS 4.2 m) (Fig. 1f-g). The patient also experienced upper abdominal pain, an upper respiratory tract infection and a severe rash. From the sixth treatment, oxaliplatin was changed to irinotecan (180 mg/m^2^ iv over 30–90 min on day 1). After the sixth treatment cycle, hypocalcemia (mean, 5.8 mg/dL; range, 4.3–8.2 mg/dL; diagnostic lower limit, 14.0 mg/dL) and a tendency of hypokalemia (mean, 142.4 mEq/L; range, 118.6–161.1 mEq/L; diagnostic lower limit, 136.5 mEq/L) were observed (Fig. 1h). They were not relieved by intravenous calcium gluconate supplementation (1 g, twice daily).

Prior to the eighth treatment, a CT examination revealed an increase in total liver mass (Fig. 1i), which indicated PD (PFS 1.3 m). Unfortunately, the condition of the patient deteriorated with severe ascites and infection. Finally, best supportive care lasted for 1 month before he died (OS was 7.5 m).

## Discussion and conclusions

Primary immunodeficiency caused by the *BTK* mutation is the main reason for XLA-associated diseases, which include recurrent bacterial infections, arthritis and types of cancer [23, 24]. In this study, the diagnosis of XLA was confirmed by WES, where a hemizygous pathogenic mutation (c.340_347del, p.F114delX115) of the *BTK* gene was found (Additional file 1). The 8 bp deletion of TTCTCCCC resulted in a frameshift mutation that affected general membrane targeting and the regulatory function of BTK [25]. Immunohistochemistry showed similar low expression of BTK in the tumor and tumor-adjacent tissue (Additional file 4: Figure S2).

It has been hypothesized that male XLA patients tend to have an early-onset CRC [11, 12]. B-cell deficiency is a risk factor for a narrow range of solid cancers, including CRC [26]. However, the correlation between CRC and XLA has not yet been statistically confirmed with a reasonable sample size. One study found colorectal adenomatous polyps from 2 out of 4 XLA patients in the Netherlands [27]. However, two other studies found that among 44 and 27 XLA patients from the United Kingdom and mainland China, respectively, none of the patients developed CRC [5, 28]. In the case studied in this report, the onset of CRC was more likely due to sporadic colorectal mutations. First, the XLA patient had no family history of immunodeficiency within 3 generations. Second, among the 10 somatic variants, the most convincing candidate CRC-driver mutations in *ABCA6, C6,* and *PAX3* provided by the DriverDBv2 database are not functionally related [29]. *C6* encodes for one of the membrane attack proteins, which plays a key role in the innate and adaptive immune responses by forming pores in the plasma membrane of target cells. It has been shown that dextran-sulfate-sodium (DSS) induced colitis was aggravated in C6-deficient mice with a series of enhanced production of pro-inflammatory mediators, including *IL-1β, IL-6, CXCL-1, CCL-3, TGF-β1* and *IL-17F*, compared with wild-type mice [30]. In addition, exogenous *C6* could ameliorate DSS-induced colitis in *C6*-deficient mice [30]. Since both colitis and enhanced inflammatory are risk factors of colorectal cancer, the deficiency of *C6* may also participate in CRC development [31]. *PAX3* encodes a transcription factor with an N-terminal DNA binding domain consisting of a paired box. It acts as a transcriptional regulator to activate or repress target genes of carcinogenesis [32, 33]. *ABCA6* is a member of the ATP-binding cassette transporter family. It is ubiquitously expressed in the liver, heart and brain, contributing to drug resistance and tumor metastasis [34, 35]. In addition to these cancer-promoting mutations, the mutation (c.857G > A) of N-acetyltransferase 2 (*NAT2*) has been shown to be associated with slow acetylator phenotypes, which is normally correlated with the effectiveness of drugs and xenobiotic toxicity [36].

Because the incidence of CRC in China has increased rapidly over the past two decades [37, 38], and XLA patients are more prone to carcinogenesis. It is suggested to begin surveillance programs on XLA patients for CRC screening, especially for patients over the age of 20 and 30. As most XLA patients with gastric cancer or CRC have been found at these ages [23].

Regarding severe hypocalcemia, it is speculated to be a joint result of a number of germline mutations in genes related to calcium metabolism and the administration of irinotecan. First, based on the enrichment analysis of genes having germline mutations, the largest enriched group was calcium-related proteins (*n* = 48, *p* = 1.2E-7) (Additional file 5 and Additional file 6: Table S2). This group contains genes that bind at least one calcium atom or proteins whose function is calcium-dependent [39–41]. Among them, 11 genes are directly involved in calcium metabolism and transportation (Table 2). Mutations in these genes may make the patient prone to abnormal calcium metabolism such as hypocalcemia. Second, the onset of hypocalcemia occurred immediately after the administration of irinotecan. Several studies have reported that hypocalcemia may be one of the rare DRTs of irinotecan (Table 3). Most of these cases had concurrent electrolyte abnormalities such as hypokalemia and hypomagnesemia. Consistently, the patient in this study also showed a trend of hypokalemia (Fig. 2j). However, the magnesium level was not measured in this study.

**Table 2:** Germline variants related to calcium metabolism

SNP ID	Gene	Chrs	Gene Region	Function	Calcium-related function or diseases
p.F114delX115	BTK	X	exonic	nonsynonymous SNV	Induces calcium mobilization and calcium-mediated signaling.
rs202054008	CACNA2D4	4	exonic	nonsynonymous SNV	Regulates calcium current density and activation/inactivation of calcium channels
rs75326924	CD36	3	exonic	nonsynonymous SNV	Regulates intracellular calcium levels by long-chain fatty acids.
rs2229291	CPT2	15	splicing	NA	Calcium metabolism and abnormal calcium deposition.
rs3888798	CTSC	X	exonic	frameshift deletion	Deposition of calcium salts in a tissue or location in which calcification does not normally occur.
rs147630160	CXCL16	10	exonic	nonsynonymous SNV	Induces calcium mobilization.
rs117643139	DRD2	4	exonic	nonsynonymous SNV	Pituitary adenoma-related hypokalemia and hypocalcemia [42]
rs139997095	PKD1L2	7	exonic	nonsynonymous SNV	Calcium ion transmembrane transport.
rs201550522	PRSS1	6	exonic	nonsynonymous SNV	Deposition of calcium salts in a tissue or location in which calcification does not normally occur.
rs201533738	SLX4	7	exonic	nonsynonymous SNV	Calcium metabolism.
rs182693954	TRPV1	12	exonic	nonsynonymous SNV	Ligand-activated nonselective calcium permeant cation channel involved in the detection of noxious chemical and thermal stimuli.

**Table 3:** Reported cases showing a positive association between irinotecan and hypocalcemia

Case	Country	Tumor	Stage	Age	Drug treatment	Serum electrolyte abnormalities	Onset time
2003 [43]	United states	Solid tumors	NA	4–21	Cisplatin, irinotecan, amifostine	Hypocalcemia	Within 24 h after 1st treatment
2005 [44, 45]	United states	Colorectal cancer	TxNxM1	34	Cetuximab, irinotecan	Hypocalcemia, hypomagnesemia	8 weeks after 1st treatment
2009 [46]	United states	Colorectal cancer	T3N2M0	77	Cap, irinotecan, bevacizumab	Hypocalcemia, hypophosphatemia, hypokalemia, hypouricemia	5 days after the 11th treatment
2010 [47]	Japan	Colorectal cancer	TxNxM1	61	Cetuximab, irinotecan	Hypocalcemia, hypomagnesemia	NA
2012 [48]	Turkey	Breast cancer	T2N0M0	57	Irinotecan, trastuzumab	Hypokalemia, hypocalcemia, hypomagnesemia	6th week of treatment

In conclusion, this study reported the first written record of a Chinese XLA patient with metastatic CRC and severe hypocalcemia following irinotecan administration. The chemotherapy regimen was carefully determined based on TDM and WES. Based on WES results and bioinformatic analysis, a germline mutation of *BTK* was confirmed to be the cause of XLA, and somatic mutations of *ABCA6, C6,* and *PAX3* may contribute to the onset and metastasis of CRC. The administration of irinotecan and a number of germline mutations on genes related to calcium metabolism might collectively cause hypocalcemia.

## Additional files

Additional file 1: Methods of DNA sequencing and screening. The detailed method used for carrying out DNA sequencing and screening was described in words with references. (DOCX 42 kb)

Additional file 2: **Figure S1.** Variant filtering pipeline for Whole Exome Sequencing. The step-by-step flow chart for whole exosome sequencing was illustrated with key nodes. (DOCX 116 kb)

Additional file 3: **Table S1.** Mutations that related to efficacy (or safety) of 5-FU-like drugs. The self-selected genes or SNVs which may affect the efficacy or safety of 5-FU-based chemotherapy were listed with references. They were sub-grouped into three types which are related to 5-FU metabolism, cancer development (oncogenes), or lipid metabolism respectively. (DOCX 202 kb)

Additional file 4: **Figure S2.** BTK expression in liver tumor tissue. BTK expression in liver tumor tissue was positively illustrated by immunohistochemistry. (DOCX 1843 kb)

Additional file 5: Germline variants illuminating hypocalcemia. Bioinformatic analysis on germline variants indicated that many of these variants were related to calcium binding and transporting, several of them might even be the main reason for the hypocalcemia. (DOCX 29 kb)

Additional file 6: **Table S2.** Germline variants related calcium binding and transporting. All of the germline line variants related to calcium binding and transporting were listed here with annotations on whether it is cancer related and its gene function. (DOCX 34 kb)

## Data Availability

The datasets used and/or analyzed during the current study are available from the corresponding author upon a reasonable request.

## Abbreviations

5′-DFUR: Doxifluridine
5-FU: 5-fluorouracil
ABCA6: ATP-Binding Cassette Subfamily A Member 6
BTK: Bruton tyrosine kinase
C6: Complement C6
Cap: Capecitabine
CRC: Colorectal cancer
DRT: Drug-related toxicities
ECOG: Eastern Cooperative Oncology Group
IVIG: Intravenous immunoglobulin
NAT2: N-acetyltransferase 2
NCCN: The National Comprehensive Cancer Network
PAX3: Paired Box 3
TDM: Therapeutic drug monitoring
TGFB1: Transcription of transforming growth factor beta 1
TYMP: Thymidine phosphorylase
WES: Whole exome sequencing
XLA: X-linked agammaglobulinemia
