# Supplementary material for: Metastatic colorectal cancer and severe hypocalcemia following irinotecan administration in a patient with X-linked agammaglobulinemia: a case report
Source: BMC Med Genet. 2019 Sep 12;20:157. doi: 10.1186/s12881-019-0880-1 (PMC6739925; doi:10.1186/s12881-019-0880-1)
Supplement: Supplementary file 1 — Methods of DNA sequencing and screening. The detailed method used for carrying out DNA sequencing and screening was described in words with references. (DOCX 42 kb) [file 12881_2019_880_MOESM1_ESM.docx]

Additional file 1

## **Methods of DNA sequencing and screening**

In order to find out the BTK-associated cancer driver gene and to seek further treatment options, whole exome sequencing (WES) was carried out using blood and tissue samples of this patient. Exome capture was carried out using the Agilent SureSelect^XT^ Human All Exon V6 kit then sequenced using the Illumina HiSeq 2500 system at an average depth of 224X for tumor tissue and 89X for peripheral blood mononuclear cells. Briefly, the paired-end sequencing reads were aligned to the human hg19 reference exome using Burrows-Wheeler Aligner (BWA)^1^, SAMtools^2^, Picard^3^ and GATK best-practice workflow^4^. Variant calls and annotation were obtained with the use of Genome Analysis Toolkit (GATK) HaplotypeCaller^5^, VarScan^6,7^ and ANNOVAR^8^ program. There were 141,095 single nucleotide variants (SNVs) and small insertions and deletions (InDels) were detected to be differed from the reference sequence, which subsequently annotated with information from several variant databases, including but not limited to Human Gene Mutation Database (HGMD)^9^, 1000 Genomes Project^10,11^, ESP6500 dataset^12^ and Exome Aggregation Consortium (ExAC)^13^.

By appropriate modification of the variant filtering pipeline suggested by Yang’s research^14^, we customized the following criteria. 1) Variants in the HGMD database with a minor allele frequency of less than 5% or none corresponding record in either the 1000 Genomes Project or the ESP6500 dataset were preserved. 2) For changes that are not in the HGMD database, synonymous variants, and common variants with minor allele frequency larger than 1% in Han Chinese in Beijing population of 1000 Genomes Project, ESP6500 dataset or East Asian population of ExAC were discarded. 3) Only variants located in the exonic regions or splicing sites were included in the focused report (Figure S1).

# References

1. Li H, Durbin R. Fast and accurate short read alignment with Burrows-Wheeler transform. *Bioinformatics*. 2009;25(14):1754-1760. doi:10.1093/bioinformatics/btp324

2. Li H, Handsaker B, Wysoker A, et al. The Sequence Alignment/Map format and SAMtools. *Bioinformatics*. 2009;25(16):2078-2079. doi:10.1093/bioinformatics/btp352

3. Picard. http://broadinstitute.github.io/picard.

4. Van der Auwera GA, Carneiro MO, Hartl C, et al. From FastQ data to high confidence variant calls: the Genome Analysis Toolkit best practices pipeline. *Curr Protoc Bioinforma*. 2013;43:11.10.1-33. doi:10.1002/0471250953.bi1110s43

5. McKenna A, Hanna M, Banks E, et al. The Genome Analysis Toolkit: A MapReduce framework for analyzing next-generation DNA sequencing data. *Genome Res*. 2010;20(9):1297-1303. doi:10.1101/gr.107524.110

6. Koboldt DC, Chen K, Wylie T, et al. VarScan: variant detection in massively parallel sequencing of individual and pooled samples. *Bioinformatics*. 2009;25(17):2283-2285. doi:10.1093/bioinformatics/btp373

7. Koboldt DC, Zhang Q, Larson DE, et al. VarScan 2: Somatic mutation and copy number alteration discovery in cancer by exome sequencing. *Genome Res*. 2012;22(3):568-576. doi:10.1101/gr.129684.111

8. Wang K, Li M, Hakonarson H. ANNOVAR: functional annotation of genetic variants from high-throughput sequencing data. *Nucleic Acids Res*. 2010;38(16). doi:10.1093/nar/gkq603

9. Stenson PD, Ball E V, Mort M, et al. Human gene mutation database (HGMD (R)): 2003 update. *Hum Mutat*. 2003;21(6):577-581. doi:10.1002/humu.10212

10. Altshuler DM, Durbin RM, Abecasis GR, et al. A global reference for human genetic variation. *Nature*. 2015;526(7571):68-+. doi:10.1038/nature15393

11. Sudmant PH, Rausch T, Gardner EJ, et al. An integrated map of structural variation in 2,504 human genomes. *Nature*. 2015;526(7571):75-+. doi:10.1038/nature15394

12. Exome Variant Server, NHLBI GO Exome Sequencing Project (ESP), Seattle, WA. http://evs.gs.washington.edu/EVS/.

13. Lek M, Karczewski KJ, Minikel E V, et al. Analysis of protein-coding genetic variation in 60,706 humans. *Nature*. 2016;536(7616):285-+. doi:10.1038/nature19057

14. Yang Y, Muzny DM, Reid JG, et al. Clinical Whole-Exome Sequencing for the Diagnosis of Mendelian Disorders. *N Engl J Med*. 2013;369(16):1502-1511. doi:10.1056/NEJMoa1306555
