# Supplementary figures and images for: Metastatic colorectal cancer and severe hypocalcemia following irinotecan administration in a patient with X-linked agammaglobulinemia: a case report
Source: BMC Med Genet. 2019 Sep 12;20:157. doi: 10.1186/s12881-019-0880-1 (PMC6739925; doi:10.1186/s12881-019-0880-1)

Additional file 2：


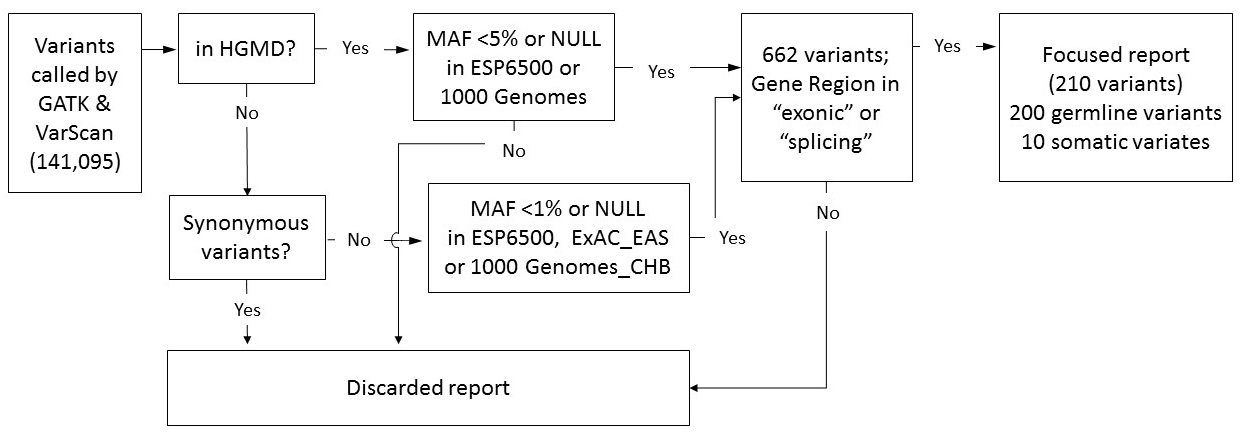


Figure S1. Variant filtering pipeline for Whole Exome Sequencing.

Supplement: Supplementary file 2 — Figure S1. Variant filtering pipeline for Whole Exome Sequencing. The step-by-step flow chart for whole exosome sequencing was illustrated with key nodes. (DOCX 116 kb) [file 12881_2019_880_MOESM2_ESM.docx]

Additional file 4


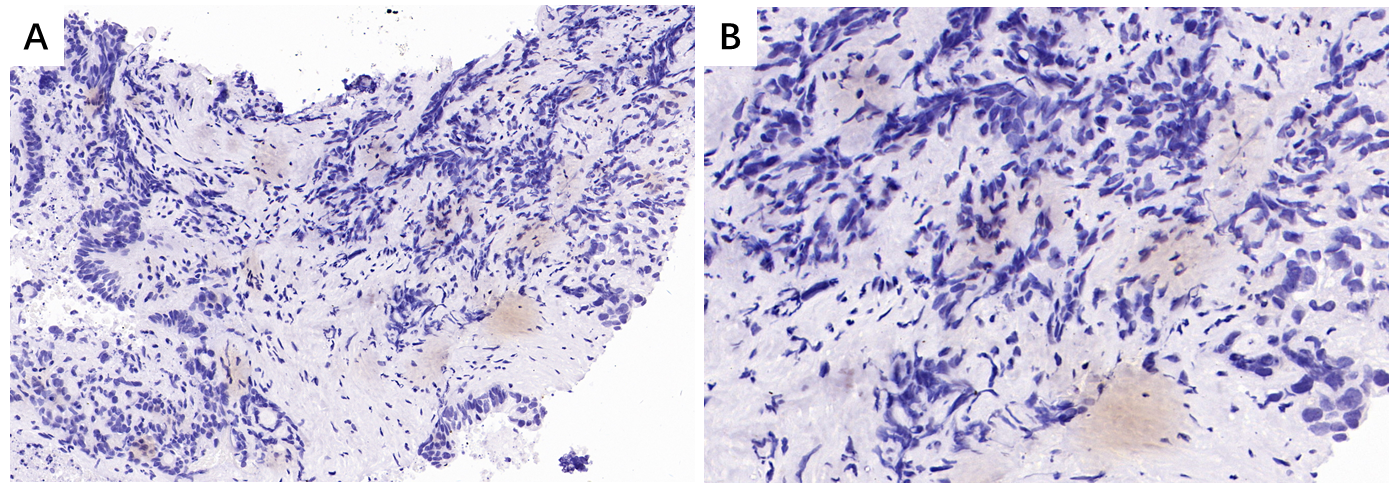


**Figure S2. BTK expression in liver tumor tissue.** (A) 20X (B) 40X

Supplement: Supplementary file 4 — Figure S2. BTK expression in liver tumor tissue. BTK expression in liver tumor tissue was positively illustrated by immunohistochemistry. (DOCX 1843 kb) [file 12881_2019_880_MOESM4_ESM.docx]
