# Supplementary material for: Metastatic colorectal cancer and severe hypocalcemia following irinotecan administration in a patient with X-linked agammaglobulinemia: a case report
Source: BMC Med Genet. 2019 Sep 12;20:157. doi: 10.1186/s12881-019-0880-1 (PMC6739925; doi:10.1186/s12881-019-0880-1)
Supplement: Supplementary file 3 — Table S1. Mutations that related to efficacy (or safety) of 5-FU-like drugs. The self-selected genes or SNVs which may affect the efficacy or safety of 5-FU-based chemotherapy were listed with references. They were sub-grouped into three types which are related to 5-FU metabolism, cancer development (oncogenes), or lipid metabolism respectively. (DOCX 202 kb) [file 12881_2019_880_MOESM3_ESM.docx]

Additional file 3

Table S1 Mutations that related to efficacy (or safety) of 5-FU-like drugs

| **Gens** | **Name** | **Mutations** | **Ref.** |
| --- | --- | --- | --- |
| Mutations related to metabolism of 5-FU and its metabolism | | | |
| ABCB1 | ATP-binding cassette, sub-family B | rs12535512 | ^1^ |
| ABCB1 | ATP-binding cassette, sub-family B | rs13233308 | ^1^ |
| ABCB1 | ATP-binding cassette, sub-family B | rs17160359 | ^2^ |
| ABCB1 | ATP-binding cassette, sub-family B | rs1922240 | ^1^ |
| ABCB1 | ATP-binding cassette, sub-family B | rs2235035 | ^1^ |
| ABCB1 | ATP-binding cassette, sub-family B | rs4148732 | ^1^ |
| ABCB1 | ATP-binding cassette, sub-family B | rs4148733 | ^1^ |
| ABCB1 | ATP-binding cassette, sub-family B | rs61122623 | ^1^ |
| ABCC3 | ATP-binding cassette, sub-family C, member 3 |  | ^1^ |
| ABCC4 | ATP-binding cassette, sub-family C, member 4 | rs1059751 | ^2^ |
| ABCC4 | ATP-binding cassette, sub-family C, member 4 | rs1479390 | ^1^ |
| ABCC4 | ATP-binding cassette, sub-family C, member 4 | rs1729788 | ^1^ |
| ABCC4 | ATP-binding cassette, sub-family C, member 4 | rs2766482 | ^1^ |
| ABCC4 | ATP-binding cassette, sub-family C, member 4 | rs3742106 | ^2^ |
| ABCC4 | ATP-binding cassette, sub-family C, member 4 | rs4148424 | ^1^ |
| ABCC4 | ATP-binding cassette, sub-family C, member 4 | rs4148432 | ^1^ |
| ABCC4 | ATP-binding cassette, sub-family C, member 4 | rs4148551 | ^2^ |
| ABCC4 | ATP-binding cassette, sub-family C, member 4 | rs7325861 | ^1^ |
| ABCC4 | ATP-binding cassette, sub-family C, member 4 | rs7986087 | ^1^ |
| ABCC4 | ATP-binding cassette, sub-family C, member 4 | rs899498 | ^1^ |
| ABCC5 | ATP-binding cassette, sub-family C, member 5 |  | ^1^ |
| ABCG2 | ATP-binding cassette, sub-family G, member 2 | rs1564481 | ^1^ |
| ABCG2 | ATP-binding cassette, sub-family G, member 2 | rs2622629 | ^1^ |
| ABCG2 | ATP-binding cassette, sub-family G, member 2 | rs2725256 | ^1^ |
| ABCG2 | ATP-binding cassette, sub-family G, member 2 | rs4693930 | ^1^ |
| CDA | cytidine deaminase |  | ^1^ |
| CES1 | carboxylesterase 1 isoform a precursor |  | ^1^ |
| CES2 | carboxylesterase 2 isoform a precursor |  | ^1^ |
| CYP2A6 | cytochrome P450 family 2 subfamily A member 6 | "CYP2A6*4C, complete deletion of the CYP2A6 gene, | ^3^ |
| CYP2A6 | cytochrome P450 family 2 subfamily A member 6 | CYP2A6*1B，conversion in the 3'-UTR | ^3^ |
| CYP2A6 | cytochrome P450 family 2 subfamily A member 6 | I471T | ^4^ |
| CYP2A6 | cytochrome P450 family 2 subfamily A member 6 | R485L | ^4^ |
| CYP2A6 | cytochrome P450 family 2 subfamily A member 6 | rs1137115 | ^5^ |
| CYP2A6 | cytochrome P450 family 2 subfamily A member 6 | rs1809810 | ^5^ |
| CYP2A6 | cytochrome P450 family 2 subfamily A member 6 | rs1809811 | ^5^ |
| CYP2A6 | cytochrome P450 family 2 subfamily A member 6 | rs2002975 | ^5^ |
| CYP2A6 | cytochrome P450 family 2 subfamily A member 6 | rs2002976 | ^5^ |
| CYP2A6 | cytochrome P450 family 2 subfamily A member 6 | rs2002977 | ^5^ |
| CYP2A6 | cytochrome P450 family 2 subfamily A member 6 | rs2644907 | ^5^ |
| CYP2A6 | cytochrome P450 family 2 subfamily A member 6 | rs28399433 | ^5^ |
| CYP2A6 | cytochrome P450 family 2 subfamily A member 6 | rs28399461 | ^5^ |
| CYP2A6 | cytochrome P450 family 2 subfamily A member 6 | rs28399468 | ^5^ |
| CYP2A6 | cytochrome P450 family 2 subfamily A member 6 | rs4079369 | ^5^ |
| CYP2A6 | cytochrome P450 family 2 subfamily A member 6 | rs5031016 | ^5^ |
| CYP2A6 | cytochrome P450 family 2 subfamily A member 6 | rs56283800 | ^5^ |
| CYP2A6 | cytochrome P450 family 2 subfamily A member 6 | rs7248240 | ^5^ |
| CYP2A6 | cytochrome P450 family 2 subfamily A member 6 | rs72549435 | ^5^ |
| CYP2A6 | cytochrome P450 family 2 subfamily A member 6 | rs8192720 | ^5^ |
| CYP2A6 | cytochrome P450 family 2 subfamily A member 6 | rs8192725 | ^5^ |
| CYP2A6 | cytochrome P450 family 2 subfamily A member 6 | rs8192726 | ^5^ |
| CYP2A6 | cytochrome P450 family 2 subfamily A member 6 | S224P | ^4^ |
| DPYD | dihydropyrimidine dehydrogenase | rs10493895 | ^2^ |
| DPYD | dihydropyrimidine dehydrogenase | rs10747486 | ^2^ |
| DPYD | dihydropyrimidine dehydrogenase | rs10783057 | ^1^ |
| DPYD | dihydropyrimidine dehydrogenase | rs10783058 | ^1^ |
| DPYD | dihydropyrimidine dehydrogenase | rs10875047 | ^1^ |
| DPYD | dihydropyrimidine dehydrogenase | rs10875061 | ^1^ |
| DPYD | dihydropyrimidine dehydrogenase | rs10875071 | ^1^ |
| DPYD | dihydropyrimidine dehydrogenase | rs10875076 | ^1^ |
| DPYD | dihydropyrimidine dehydrogenase | rs1112314 | ^1^ |
| DPYD | dihydropyrimidine dehydrogenase | rs11165779 | ^1^ |
| DPYD | dihydropyrimidine dehydrogenase | rs11165784 | ^1^ |
| DPYD | dihydropyrimidine dehydrogenase | rs11165827 | ^1^ |
| DPYD | dihydropyrimidine dehydrogenase | rs11165837 | ^1^ |
| DPYD | dihydropyrimidine dehydrogenase | rs11165845 | ^1^ |
| DPYD | dihydropyrimidine dehydrogenase | rs11165846 | ^1^ |
| DPYD | dihydropyrimidine dehydrogenase | rs11165875 | ^1^ |
| DPYD | dihydropyrimidine dehydrogenase | rs11799399 | ^1^ |
| DPYD | dihydropyrimidine dehydrogenase | rs12021567 | ^1^ |
| DPYD | dihydropyrimidine dehydrogenase | rs12022243 | ^1^ |
| DPYD | dihydropyrimidine dehydrogenase | rs12028565 | ^1^ |
| DPYD | dihydropyrimidine dehydrogenase | rs12031561 | ^1^ |
| DPYD | dihydropyrimidine dehydrogenase | rs12039249 | ^1^ |
| DPYD | dihydropyrimidine dehydrogenase | rs12040763 | ^1^ |
| DPYD | dihydropyrimidine dehydrogenase | rs12043125 | ^1^ |
| DPYD | dihydropyrimidine dehydrogenase | rs12047910 | ^1^ |
| DPYD | dihydropyrimidine dehydrogenase | rs12123160 | ^1^ |
| DPYD | dihydropyrimidine dehydrogenase | rs12132152 | ^1^ |
| DPYD | dihydropyrimidine dehydrogenase | rs12563828 | ^1^ |
| DPYD | dihydropyrimidine dehydrogenase | rs12566907 | ^1^ |
| DPYD | dihydropyrimidine dehydrogenase | rs12726453 | ^1^ |
| DPYD | dihydropyrimidine dehydrogenase | rs1333717 | ^2^ |
| DPYD | dihydropyrimidine dehydrogenase | rs1356917 | ^1^ |
| DPYD | dihydropyrimidine dehydrogenase | rs1415683 | ^1^ |
| DPYD | dihydropyrimidine dehydrogenase | rs1609519 | ^1^ |
| DPYD | dihydropyrimidine dehydrogenase | rs1709409 | ^1^ |
| DPYD | dihydropyrimidine dehydrogenase | rs1760217 | ^1^ |
| DPYD | dihydropyrimidine dehydrogenase | rs1801158 | ^1^ |
| DPYD | dihydropyrimidine dehydrogenase | rs1801159 | ^1^ |
| DPYD | dihydropyrimidine dehydrogenase | rs1801160 | ^6^ |
| DPYD | dihydropyrimidine dehydrogenase | rs1801265 | ^1^ |
| DPYD | dihydropyrimidine dehydrogenase | rs1801265 | ^6^ |
| DPYD | dihydropyrimidine dehydrogenase | rs1879375 | ^1^ |
| DPYD | dihydropyrimidine dehydrogenase | rs1931063 | ^2^ |
| DPYD | dihydropyrimidine dehydrogenase | rs2297595 | ^1^ |
| DPYD | dihydropyrimidine dehydrogenase | rs2297595 | ^7^ |
| DPYD | dihydropyrimidine dehydrogenase | rs3918290 | ^1^ |
| DPYD | dihydropyrimidine dehydrogenase | rs3918290 | ^8^ |
| DPYD | dihydropyrimidine dehydrogenase | rs4434871 | ^1^ |
| DPYD | dihydropyrimidine dehydrogenase | rs4495747 | ^1^ |
| DPYD | dihydropyrimidine dehydrogenase | rs4497250 | ^1^ |
| DPYD | dihydropyrimidine dehydrogenase | rs4537601 | ^2^ |
| DPYD | dihydropyrimidine dehydrogenase | rs45589337 | ^1^ |
| DPYD | dihydropyrimidine dehydrogenase | rs4949952 | ^1^ |
| DPYD | dihydropyrimidine dehydrogenase | rs4970728 | ^2^ |
| DPYD | dihydropyrimidine dehydrogenase | rs507170 | ^1^ |
| DPYD | dihydropyrimidine dehydrogenase | rs526645 | ^1^ |
| DPYD | dihydropyrimidine dehydrogenase | rs528455 | ^1^ |
| DPYD | dihydropyrimidine dehydrogenase | rs553388 | ^1^ |
| DPYD | dihydropyrimidine dehydrogenase | rs55886062 | ^1^ |
| DPYD | dihydropyrimidine dehydrogenase | rs55886062 | ^7^ |
| DPYD | dihydropyrimidine dehydrogenase | rs56038477 | ^1^ |
| DPYD | dihydropyrimidine dehydrogenase | rs628959 | ^1^ |
| DPYD | dihydropyrimidine dehydrogenase | rs641805 | ^1^ |
| DPYD | dihydropyrimidine dehydrogenase | rs644428 | ^1^ |
| DPYD | dihydropyrimidine dehydrogenase | rs6593642 | ^1^ |
| DPYD | dihydropyrimidine dehydrogenase | rs6678858 | ^1^ |
| DPYD | dihydropyrimidine dehydrogenase | rs6683957 | ^2^ |
| DPYD | dihydropyrimidine dehydrogenase | rs67373796 | ^1^ |
| DPYD | dihydropyrimidine dehydrogenase | rs67376798 | ^1^ ^7^ |
| DPYD | dihydropyrimidine dehydrogenase | rs7522938 | ^1^ |
| DPYD | dihydropyrimidine dehydrogenase | rs7540201 | ^1^ |
| DPYD | dihydropyrimidine dehydrogenase | rs7548189 | ^1^ |
| DPYD | dihydropyrimidine dehydrogenase | rs7550959 | ^1^ |
| DPYD | dihydropyrimidine dehydrogenase | rs7556439 | ^1^ |
| DPYD | dihydropyrimidine dehydrogenase | rs76387818 | ^1^ |
| DPYD | dihydropyrimidine dehydrogenase | rs9782950 | ^1^ |
| DPYS | dihydropyrimidinase | rs2853151 | ^1^ |
| ENOSF1 | enolase superfamily member 1 | rs11081251 | ^1^ |
| ENOSF1 | enolase superfamily member 1 | rs11873007 | ^1^ |
| ENOSF1 | enolase superfamily member 1 | rs2612086 | ^9^ |
| ENOSF1 | enolase superfamily member 1 | rs2847154 | ^1^ |
| ENOSF1 | enolase superfamily member 1 | rs3786355 | ^1^ |
| ENOSF1 | enolase superfamily member 1 | rs3819101 | ^1^ |
| MTHFR | methylenetetrahydrofolate reductase | rs1801131 | ^5,10,11^ |
| MTHFR | methylenetetrahydrofolate reductase | rs1801133 | ^5,10,11^ |
| PPAT | phosphoribosyl pyrophosphate amidotransferase |  | ^5,10^ |
| RRM1 | ribonucleoside-diphosphate reductase subunit 1 | rs1042858 | ^2^ |
| RRM1 | ribonucleoside-diphosphate reductase subunit 1 | rs1042927 | ^2^ |
| RRM1 | ribonucleoside-diphosphate reductase subunit 1 | rs1561876 | ^2^ |
| RRM1 | ribonucleoside-diphosphate reductase subunit 1 | rs1662162 | ^2^ |
| RRM1 | ribonucleoside-diphosphate reductase subunit 1 | rs1735068 | ^2^ |
| RRM1 | ribonucleoside-diphosphate reductase subunit 1 | rs3750996 | ^2^ |
| RRM1 | ribonucleoside-diphosphate reductase subunit 1 | rs3794050 | ^2^ |
| RRM1 | ribonucleoside-diphosphate reductase subunit 1 | rs7934581 | ^2^ |
| RRM2 | ribonucleoside-diphosphate reductase subunit 2 |  | ^1^ |
| SLC22A7 | solute carrier family 22 member 7 isoform b |  | ^1^ |
| SLC29A1 | equilibrative nucleoside transporter 1 |  | ^1^ |
| TK1 | thymidine kinase 1 | rs8071253 | ^2^ |
| TSER | thymidylate synthase gene enhancer region |  | ^12^ |
| TYMP | thymidine phosphorylase |  | ^1^ |
| TYMP | thymidine phosphorylase |  | ^1^ |
| TYMP | thymidine phosphorylase | rs11479 | ^13^ |
| TYMS | thymidylate synthetase | rs16430 | ^13^ |
| TYMS | thymidylate synthetase | rs2244500 | ^1^ |
| TYMS | thymidylate synthetase | rs2612091 | ^1^ |
| TYMS | thymidylate synthetase | rs2741171 | ^1^ |
| TYMS | thymidylate synthetase | rs2853542 | ^2^ |
| TYMS | thymidylate synthetase | rs45445694 | ^14^ |
| UCK1 | uridine-cytidine kinase 1 isoform a |  | ^1,10^ |
| UCK2 | uridine-cytidine kinase 2 isoform a |  | ^1^ |
| UMPS | uridine monophosphate synthase | rs12492095 | ^2^ |
| UMPS | uridine monophosphate synthase | rs1801019 | ^2^ |
| UMPS | uridine monophosphate synthase | rs2291078 | ^2^ |
| UMPS | uridine monophosphate synthase | rs3772809 | ^2^ |
| UMPS | uridine monophosphate synthase | rs3772810 | ^2^ |
| UPB1 | beta-ureidopropionase | rs2232861 | ^2^ |
| UPP1 | uridine phosphorylase 1 |  | ^1,10^ |
| UPP2 | uridine phosphorylase 2 |  | ^1^ |
| Mutations related to lipid metabolism | | | |
| ACSL5 | Acyl-CoA Synthetase Long-Chain Family Member 5 | rs17129748 | ^15^ |
| ACSL5 | Acyl-CoA Synthetase Long-Chain Family Member 5 | rs2419629 | ^15^ |
| ACSL5 | Acyl-CoA Synthetase Long-Chain Family Member 5 | rs7919710 | ^15^ |
| APOE | apolipoprotein E | rs405509 | ^15^ |
| APOE | apolipoprotein E | rs439401 | ^15^ |
| APOE | apolipoprotein E | rs7259620 | ^15^ |
| CYP1A2 | Cytochrome P450 Family 1 Subfamily A Member 2 | rs2470890 | ^15^ |
| CYP1A2 | Cytochrome P450 Family 1 Subfamily A Member 2 | rs762551 | ^15^ |
| CYP2C9 | Cytochrome P450 Family 2 Subfamily C Member 9 | rs1057910e | ^15^ |
| LIPC | hepatic lipase | C-514T | ^15^ |
| LIPC | hepatic lipase | rs12913969 | ^15^ |
| LIPC | hepatic lipase | rs16940302 | ^15^ |
| LIPC | hepatic lipase | rs16940372 | ^15^ |
| LIPC | hepatic lipase | rs17190510 | ^15^ |
| LIPC | hepatic lipase | rs1800588 | ^15^ |
| LIPC | hepatic lipase | rs2099190 | ^15^ |
| LIPC | hepatic lipase | rs3751542 | ^15^ |
| LIPC | hepatic lipase | rs4774302 | ^15^ |
| LIPC | hepatic lipase | rs4775053 | ^15^ |
| LIPC | hepatic lipase | rs4775072 | ^15^ |
| LIPC | hepatic lipase | rs6083 | ^15^ |
| LIPC | hepatic lipase | rs634746 | ^15^ |
| LIPC | hepatic lipase | rs7166788 | ^15^ |
| LIPC | hepatic lipase | rs7174210 | ^15^ |
| LIPC | hepatic lipase | rs8035006 | ^15^ |
| LIPC | hepatic lipase | rs9652472 | ^15^ |
| LPL | lipoprotein lipase | rs268 | ^15^ |
| LPL | lipoprotein lipase | rs328 | ^15^ |
| PPARG | Peroxisome Proliferator Activated Receptor Gamma | rs1801282 | ^15^ |
| PPARG | Peroxisome Proliferator Activated Receptor Gamma | rs1801282 | ^15^ |
| PPARG | Peroxisome Proliferator Activated Receptor Gamma | rs3856806 | ^15^ |
| PTGS2 | Prostaglandin-Endoperoxide Synthase 2 | rs5275 | ^15^ |
|  | | | |
| Mutations of oncogens | | | |
| ABL1-F359V | 窗体顶端  ABL proto-oncogene 1, non-receptor tyrosine kinase窗体底端 | c.1075T>G | ^16^ |
| AKT1 | AKT Serine/Threonine Kinase 1 |  | ^7,16^ |
| AKT1-E17K | 窗体顶端  AKT serine/threonine kinase 1窗体底端 | rs121434592 | ^16^ |
| APC | adenomatosis polyposis coli, WNT signaling pathway regulator | rs2431238 | ^2^ |
| APC | adenomatosis polyposis coli, WNT signaling pathway regulator | rs2464805 | ^2^ |
| APC | adenomatosis polyposis coli, WNT signaling pathway regulator | rs459552 | ^2^ |
| ATP7A | ATPase copper transporting alpha | rs1062472 | ^15^ |
| ATP7A | ATPase copper transporting alpha | rs17139617 | ^2^ |
| ATP7A | ATPase copper transporting alpha | rs2227291 | ^1^ |
| BRAF | B-Raf Proto-Oncogene, Serine/Threonine Kinase | rs113488022 | ^16^ |
| BRAF | B-Raf Proto-Oncogene, Serine/Threonine Kinase | rs121913338 | ^16^ |
| CDC2 | cyclin dependent kinase 1 | rs2448341 | ^2^ |
| CYP19A1 | cytochrome P450 family 19 subfamily A member 1 | rs2236722 | ^2^ |
| DPC4 | CG7287 gene product from transcript CG7287-RA | 全长/12bp-indel | ^17^ |
| DLG5 | discs large MAGUK scaffold protein 5 | rs2289310 | ^2^ |
| ERCC6 | excision repair 6, chromatin remodeling factor | rs4253101 | ^2^ |
| EXO1 | exonuclease 1 | rs1047840 | ^2^ |
| HTR3D | 5-hydroxytryptamine receptor 3D | rs36092077 | ^1^ |
| ITGB5 | integrin subunit beta 5 | rs2291081 | ^1^ |
| ITGB5 | integrin subunit beta 5 | rs3821536 | ^1^ |
| KRAS | KRAS Proto-Oncogene, GTPase | "KRAS mutations (including codon 61, excluding codon 146) | ^18^ |
| KRAS | KRAS Proto-Oncogene, GTPase | codon 12 and 13 mutations | ^18^ |
| KRAS | KRAS Proto-Oncogene, GTPase | Gly12Ala | ^19^ |
| KRAS | KRAS Proto-Oncogene, GTPase | Gly12Asp | ^19^ |
| KRAS | KRAS Proto-Oncogene, GTPase | Gly12Cys | ^19^ |
| KRAS | KRAS Proto-Oncogene, GTPase | Gly12Ser | ^19^ |
| KRAS | KRAS Proto-Oncogene, GTPase | Gly12Val | ^19^ |
| KRAS | KRAS Proto-Oncogene, GTPase | Gly13Asp | ^19^ |
| KRAS | KRAS Proto-Oncogene, GTPase | rs121913528 | ^16^ |
| KRAS | KRAS Proto-Oncogene, GTPase | rs121913529 | ^16^ |
| KRAS | KRAS Proto-Oncogene, GTPase | rs121913530 | ^16^ |
| KRAS | KRAS Proto-Oncogene, GTPase | rs121913529 | ^16^ |
| KRAS | KRAS Proto-Oncogene, GTPase | rs121913530 | ^16^ |
| KRAS | KRAS Proto-Oncogene, GTPase | rs121913250 | ^16^ |
| KRAS | KRAS Proto-Oncogene, GTPase | rs121913529 | ^16^ |
| KRAS | KRAS Proto-Oncogene, GTPase | rs112445441 | ^16^ |
| KRAS | KRAS Proto-Oncogene, GTPase | rs121913240 | ^16^ |
| KRAS | KRAS Proto-Oncogene, GTPase | rs121913240 | ^16^ |
| LMF2 | lipase maturation factor 2 | rs9616787 | ^1^ |
| MET | MET proto-oncogene, receptor tyrosine kinase | rs34589476 | ^16^ |
| MET | MET proto-oncogene, receptor tyrosine kinase | rs56391007 | ^16^ |
| NRAS | neuroblastoma RAS viral oncogene homolog | rs121913250 | ^16^ |
| NRAS | neuroblastoma RAS viral oncogene homolog | rs121913237 | ^16^ |
| NRAS | neuroblastoma RAS viral oncogene homolog | rs121434595 | ^16^ |
| NRAS | neuroblastoma RAS viral oncogene homolog | rs121913535 | ^16^ |
| NRAS | neuroblastoma RAS viral oncogene homolog | rs121913255 | ^16^ |
| NRAS | neuroblastoma RAS viral oncogene homolog | rs121913254 | ^16^ |
| PIK3CA | phosphatidylinositol-4,5-bisphosphate 3-kinase catalytic subunit alpha | exon 20 mutations | ^18^ |
| PIK3CA | phosphatidylinositol-4,5-bisphosphate 3-kinase catalytic subunit alpha | rs121913272 | ^16^ |
| PIK3CA | phosphatidylinositol-4,5-bisphosphate 3-kinase catalytic subunit alpha | rs121913273 | ^16^ |
| PIK3CA | phosphatidylinositol-4,5-bisphosphate 3-kinase catalytic subunit alpha | rs104886003 | ^16^ |
| PIK3CA | phosphatidylinositol-4,5-bisphosphate 3-kinase catalytic subunit alpha | rs121913279 | ^16^ |
| PIK3CA | phosphatidylinositol-4,5-bisphosphate 3-kinase catalytic subunit alpha | rs121913279 | ^16^ |
| PIK3CA | phosphatidylinositol-4,5-bisphosphate 3-kinase catalytic subunit alpha | c.2102A>C | ^16^ |
| PIK3CA | phosphatidylinositol-4,5-bisphosphate 3-kinase catalytic subunit alpha | rs121913286 | ^16^ |
| PIK3CA | phosphatidylinositol-4,5-bisphosphate 3-kinase catalytic subunit alpha | c.263G>A | ^16^ |
| POMT1 | protein O-mannosyltransferase 1 | rs147266709 | ^1^ |
| POMT1 | protein O-mannosyltransferase 1 | rs2651204 | ^1^ |
| REV3L | REV3 like, DNA directed polymerase zeta catalytic subunit | rs3218592 | ^2^ |
| SLCO6A1 | solute carrier organic anion transporter family member 6A1 | rs10041507 | ^2^ |
| SLCO6A1 | solute carrier organic anion transporter family member 6A1 | rs10041525 | ^2^ |
| SLCO6A1 | solute carrier organic anion transporter family member 6A1 | rs10062613 | ^2^ |
| SLCO6A1 | solute carrier organic anion transporter family member 6A1 | rs11746217 | ^2^ |
| SLCO6A1 | solute carrier organic anion transporter family member 6A1 | rs1452057 | ^2^ |
| SLCO6A1 | solute carrier organic anion transporter family member 6A1 | rs1562961 | ^2^ |
| SLCO6A1 | solute carrier organic anion transporter family member 6A1 | rs1901512 | ^2^ |
| SLCO6A1 | solute carrier organic anion transporter family member 6A1 | rs1901521 | ^2^ |
| SLCO6A1 | solute carrier organic anion transporter family member 6A1 | rs1901522 | ^2^ |
| SLCO6A1 | solute carrier organic anion transporter family member 6A1 | rs6873738 | ^2^ |
| SLCO6A1 | solute carrier organic anion transporter family member 6A1 | rs6877722 | ^2^ |
| SMARCA2 | SWI/SNF related, matrix associated, actin dependent regulator of chromatin, subfamily a, member 2 | rs7048976 | ^2^ |
| TFRC | transferrin receptor | rs3817672 | ^2^ |
| WDR7 | WD repeat domain 7 | rs11664579 | ^2^ |
| WDR7 | WD repeat domain 7 | rs11876256 | ^2^ |
| WDR7 | WD repeat domain 7 | rs11877604 | ^2^ |
| WDR7 | WD repeat domain 7 | rs2083020 | ^2^ |
| WDR7 | WD repeat domain 7 | rs2307083 | ^2^ |
| WDR7 | WD repeat domain 7 | rs2576415 | ^2^ |
| WDR7 | WD repeat domain 7 | rs3745030 | ^2^ |
| WDR7 | WD repeat domain 7 | rs3745032 | ^2^ |
| WDR7 | WD repeat domain 7 | rs501415 | ^2^ |
| WDR7 | WD repeat domain 7 | rs6566846 | ^2^ |
| WDR7 | WD repeat domain 7 | rs8094838 | ^2^ |
| WDR7 | WD repeat domain 7 | rs9946253 | ^2^ |

# References

1. Rosmarin D, Palles C, Pagnamenta A, et al. A candidate gene study of capecitabine-related toxicity in colorectal cancer identifies new toxicity variants at DPYD and a putative role for ENOSF1 rather than TYMS. *Gut*. 2015;64(1):111-120. doi:10.1136/gutjnl-2013-306571

2. Wang J, Wang X, Zhao M, et al. Potentially functional SNPs (pfSNPs) as novel genomic predictors of 5-FU response in metastatic colorectal cancer patients. *PLoS One*. 2014;9(11). doi:10.1371/journal.pone.0111694

3. Fang WJ, Mou HB, Jin DZ, et al. Characteristic CYP2A6 genetic polymorphisms detected by TA cloning-based sequencing in Chinese digestive system cancer patients with S-1 based chemotherapy. *Oncol Rep*. 2012;27(5):1606-1610. doi:10.3892/or.2012.1678

4. Yamamiya I, Yoshisue K, Ishii Y, Yamada H, Chiba M. Effect of CYP2A6 genetic polymorphism on the metabolic conversion of tegafur to 5-fluorouracil and its enantioselectivity. *Drug Metab Dispos*. 2014;42(9):1485-1492. doi:10.1124/dmd.114.058008

5. Wang H, Bian T, Liu D, et al. Association analysis of CYP2A6 genotypes and haplotypes with 5-fluorouracil formation from tegafur in human liver microsomes. *Pharmacogenomics*. 2011;12(4):481-492. doi:10.2217/pgs.10.202

6. Zhang X, Sun B, Lu Z. Evaluation of clinical value of single nucleotide polymorphisms of dihydropyrimidine dehydrogenase gene to predict 5-fluorouracil toxicity in 60 colorectal cancer patients in China. *Int J Med Sci*. 2013;10(7):894-902. doi:10.7150/ijms.5556

7. Kleist B, Kempa M, Meurer T, Poetsch M. Correlation between DPYD gene variation and KRAS wild type status in colorectal cancer. 2016;9700:204-208. doi:10.1136/jclinpath-2015-202903

8. Deenen MJ, Meulendijks D, Cats A, et al. Upfront genotyping of DPYD*2A to individualize fluoropyrimidine therapy: a safety and cost analysis. *J Clin Oncol*. 2016;34(3):Under consideration. doi:10.1200/JCO.2015.63.1325

9. Huang L, Chen F, Chen Y, et al. Thymidine phosphorylase gene variant, platelet counts and survival in gastrointestinal cancer patients treated by fluoropyrimidines. *Sci Rep*. 2014;4(1):5697. doi:10.1038/srep05697

10. Negrei C, Hudita A, Ginghina O, et al. Colon Cancer Cells Gene Expression Signature As Response to 5- Fluorouracil, Oxaliplatin, and Folinic Acid Treatment. *Front Pharmacol*. 2016;7(June):172. doi:10.3389/fphar.2016.00172

11. Loganayagam A, Arenas Hernandez M, Corrigan A, et al. Pharmacogenetic variants in the DPYD, TYMS, CDA and MTHFR genes are clinically significant predictors of fluoropyrimidine toxicity. *Br J Cancer*. 2013;108(12):2505-2515. doi:10.1038/bjc.2013.262

12. Soo RA, Syn N, Lee S, et al. Pharmacogenetics-Guided Phase I Study of Capecitabine on an Intermittent Schedule in Patients with Advanced or Metastatic Solid Tumours. *Nat Publ Gr*. 2016;(May):1-10. doi:10.1038/srep27826

13. Rosmarin D, Palles C, Church D, et al. Genetic markers of toxicity from capecitabine and other fluorouracil-based regimens: Investigation in the QUASAR2 study, systematic review, and meta-analysis. *J Clin Oncol*. 2014;32(10):1031-1039. doi:10.1200/JCO.2013.51.1857

14. Hammad H, Sarkar M, Gupta N, Ardalan B, Subbarayan PR. The presence of three repeats in the 5’UTR region of thymidylate synthase (TS) is associated with increased TS mRNA expression in cultured human cancer cell lines in vitro. *Oncol Rep*. 2012;27(1):246-249. doi:10.3892/or.2011.1469

15. Crous-Bou M, Rennert G, Salazar R, et al. Genetic polymorphisms in fatty acid metabolism genes and colorectal cancer. *Mutagenesis*. 2012;27(2):169-176. doi:10.1093/mutage/ger066

16. Fumagalli D, Gavin PG, Taniyama Y, et al. A rapid, sensitive, reproducible and cost-effective method for mutation profiling of colon cancer and metastatic lymph nodes. *BMC Cancer*. 2010;10:101. doi:10.1186/1471-2407-10-101

17. YUKIHIRO TAKAGI,* HISASHI KOHMURA,* MANABU FUTAMURA,* HISASHI KIDA,* HIROMI TANEMURA KS, SAJI* and S. Somatic Alterations of the DPC4 Gene in Human Colorectal Cancers In Vivo. 1996;111(4):746-751. doi:10.1525/auk.2010.10132

18. Roock W De, Vriendt V De, Normanno N, Ciardiello F, Tejpar S. KRAS, BRAF, PIK3CA, and PTEN mutations: Implications for targeted therapies in metastatic colorectal cancer. *Lancet Oncol*. 2011;12(6):594-603. doi:10.1016/S1470-2045(10)70209-6

19. Cejas P, López-Gómez M, Aguayo C, et al. KRAS mutations in primary colorectal cancer tumors and related metastases: A potential role in prediction of lung metastasis. *PLoS One*. 2009;4(12). doi:10.1371/journal.pone.0008199
