# Supplementary material for: Metastatic colorectal cancer and severe hypocalcemia following irinotecan administration in a patient with X-linked agammaglobulinemia: a case report
Source: BMC Med Genet. 2019 Sep 12;20:157. doi: 10.1186/s12881-019-0880-1 (PMC6739925; doi:10.1186/s12881-019-0880-1)
Supplement: Supplementary file 5 — Germline variants illuminating hypocalcemia. Bioinformatic analysis on germline variants indicated that many of these variants were related to calcium binding and transporting, several of them might even be the main reason for the hypocalcemia. (DOCX 29 kb) [file 12881_2019_880_MOESM5_ESM.docx]

Additional file 5

## **Germline mutations illuminating hypocalcemia**

| In order to find out genes that may responsible for the hypocalcemia developed during chemotherapy, 200 selected germline SNVs/InDels were further annotated by David^1,2^ and VarElect^3^. Amongst all of the gene functional annotation categories, the most enriched gene group is the calcium related proteins, which binds at least one calcium atom, or protein whose function is calcium-dependent (p = 1.2E-7). A number of 48 identified germline SNVs/InDels were belong to this group, which constitute 24% of the total number (Table S3). Referencing annotations from variant databases of these 48 SNVs/InDels, 4 of them were predicted to be the most possible candidates and listed in front. According to Gene Ontology (GO) molecular function annotations, PCDH15, NELL1 and WFS1 are all connected to calcium ion binding, while DRD2 regulates ion homeostasis of both calcium and potassium.  Based on the correlation with hypocalcemia by DisGeNET (http://www.disgenet.org), germline mutations in GNAS and BTK were also potential causes of hypocalcemia. BTK expression showed no difference between tumor and stroma (Figure S2), probably owning to inborn pathogenic BTK mutation and following extremely low protein level of the patient. |  |  |
| --- | --- | --- |

# References

1. Huang DW, Sherman BT, Lempicki RA. Bioinformatics enrichment tools: paths toward the comprehensive functional analysis of large gene lists. *Nucleic Acids Res*. 2009;37(1):1-13. doi:10.1093/nar/gkn923

2. Huang DW, Sherman BT, Lempicki RA. Systematic and integrative analysis of large gene lists using DAVID bioinformatics resources. *Nat Protoc*. 2009;4(1):44-57. doi:10.1038/nprot.2008.211

3. Stelzer G, Plaschkes I, Oz-Levi D, et al. VarElect: the phenotype-based variation prioritizer of the GeneCards Suite. *BMC Genomics*. 2016;17. doi:10.1186/s12864-016-2722-2
