# Supplementary material for: Metastatic colorectal cancer and severe hypocalcemia following irinotecan administration in a patient with X-linked agammaglobulinemia: a case report
Source: BMC Med Genet. 2019 Sep 12;20:157. doi: 10.1186/s12881-019-0880-1 (PMC6739925; doi:10.1186/s12881-019-0880-1)
Supplement: Supplementary file 6 — Table S2. Germline variants related calcium binding and transporting. All of the germline line variants related to calcium binding and transporting were listed here with annotations on whether it is cancer related and its gene function. (DOCX 34 kb) [file 12881_2019_880_MOESM6_ESM.docx]

Additional file 6

Table S2 Shared variants related calcium binding and transporting

| **Gene** | **Mutation** | **Related to Cancers?** | **Calcium-related Gene function** |
| --- | --- | --- | --- |
| PCDH15 | rs201816080 | Yes | Calcium-dependent cell-adhesion protein. |
| NELL1 | rs201961389 | Yes | EGF-like calcium-binding. |
| WFS1 | rs1801208 | - | Participates in the regulation of cellular Ca(2+) homeostasis, at least partly, by modulating the filling state of the endoplasmic reticulum Ca(2+) store. |
| DRD2 | rs1801028 | - | Related to Pituitary Adenoma, whose symptoms include hypokalemia and hypocalcemia. |
| MATN4 | rs117643139 | Yes | Calcium ion binding. |
| CBLB | rs200880425 | Yes | Calcium ion binding. |
| CDH23 | rs74145660 | Yes | Cadherin-related 23. Cadherins are calcium-dependent cell adhesion proteins. |
| CXCL16 | rs147630160 | Yes | Induces calcium mobilization. |
| MMP25 | rs192194204 | Yes | Calcium ion binding. |
| SHH | rs104894047 | Yes | Calcium ion binding. |
| THBS1 | rs185847032 | Yes | Calcium ion binding. |
| PLCD1 | rs933135 | Yes | Calcium ion binding. |
| ARSH | rs201825434 | Yes | Drugs for DNASE1 gene include calcium element. |
| MMP7 | rs17884789 | Yes | Drugs for DNASE1 gene include calcium element. |
| SPON2 | rs200308601 | Yes | The protein structure revealed an eight-stranded antiparallel beta-sandwich motif resembling that of membrane-targeting C2 domains, including a bound calcium ion. |
| CACNA2D4 | rs202054008 | - | The alpha-2/delta subunit of voltage-dependent calcium channels regulates calcium current density and activation/inactivation kinetics of the calcium channel. |
| TRPV1 | rs182693954 | - | Ligand-activated non-selective calcium permeant cation channel involved in detection of noxious chemical and thermal stimuli. |
| CDH24 | rs201413160 | - | Cadherin 24, type 2. Cadherins are calcium-dependent cell adhesion proteins. |
| CDHR2 | rs115404865 | - | Intermicrovillar adhesion molecule that forms, via its extracellular domain, calcium-dependent heterophilic complexes with CDHR5 on adjacent microvilli. |
| BEST1 | rs765333778 | - | Forms calcium-sensitive chloride channels. |
| FBN1 | rs200368037 | - | Fibrillin 1. Fibrillins are structural components of 10-12 nm extracellular calcium-binding microfibrils, which occur either in association with elastin or in elastin-free bundles. |
| CPT2 | rs2229291 | - | Calcium metabolism. Abnormal calcium deposition. |
| F12 | rs183643295 | - | Calcium ion binding. |
| PLXNC1 | rs138337903 | - | Plexin C1, calcium-dependent cell adhesion molecule at the neuronal cell surface,receptor for the semaphorin 7A. |
| SCN5A | rs41261344 | - | It is a tetrodotoxin-resistant Na(+) channel isoform. Channel inactivation is regulated by intracellular calcium levels. |
| CD36 | rs75326924 | - | In taste receptor cells, mediates the induction of an increase in intracellular calcium levels by long-chain fatty acids. |
| PKD1L2 | rs139997095 | - | Calcium ion transmembrane transport. |
| HSD17B4 | rs150326995 | - | Deposition of calcium salts in a tissue or location in which calcification does not normally occur. |
| F5 | rs201768061 | - | The protein is composed of a heavy chain and a light chain. The interaction between the two chains is calcium-dependent. |
| TTN | rs192127273 | - | Calcium ion binding. |
| HMCN1 | rs184081240 | - | Calcium ion binding. |
| MACF1 | rs181850215 | - | Calcium ion binding. |
| DNASE1 | rs77254040 | - | Drugs for DNASE1 gene include calcium element. |
| GALNS | rs78127134 | - | Drugs for DNASE1 gene include calcium element. |
| BTK | p.F114fs | - | Induces calcium mobilization. Calcium-mediated signaling. |
| CTSC | rs3888798 | Yes | Deposition of calcium salts in a tissue or location in which calcification does not normally occur. |
| BCL10 | rs12037217 | Yes | Bcl10 is phosphorylated on Ser138 by Ca2+/calmodulin-dependent protein kinase II. |
| CGREF1 | rs1057391 | Yes | Calcium ion binding. |
| FBN1 | rs140598 | - | Fibrillin 1. Fibrillins are structural components of 10-12 nm extracellular calcium-binding microfibrils, which occur either in association with elastin or in elastin-free bundles. |
| GUCY2D | rs34598902 | - | Activated by GCAP-1; inhibited by calcium. |
| PRKN | rs1801474 | Yes | May play a role in controlling neurotransmitter trafficking at the presynaptic terminal and in calcium-dependent exocytosis. |
| PRSS1 | rs201550522 | Yes | Deposition of calcium salts in a tissue or location in which calcification does not normally occur. |
| SLX4 | rs201533738 | - | Calcium metabolism. |
| F5 | rs9332608 | Yes | The protein is composed of a heavy chain and a light chain. The interaction between the two chains is calcium-dependent. |
| TTN | rs188395969 | - | Calcium ion binding. |
| FLG | rs145675213 | - | Calcium ion binding. |
| ITGA7 | rs113651939 | - | Interacts (via C-terminus intracellular tail region) with CIB1. The interaction is stabilized/increased in a calcium- and magnesium-dependent manner. |
| CYP11B2 | rs4545 | Yes | Calcium regulates human CYP11B2 transcription. |
